# Molecular mechanisms of neuronal death in brain injury after subarachnoid hemorrhage

**DOI:** 10.3389/fncel.2022.1025708

**Published:** 2022-12-13

**Authors:** Junhui Chen, Mingchang Li, Zhuanghua Liu, Yuhai Wang, Kun Xiong

**Affiliations:** ^1^Department of Human Anatomy and Neurobiology, School of Basic Medical Science, Central South University, Changsha, Hunan, China; ^2^Department of Neurosurgery, Renmin Hospital of Wuhan University, Wuhan, China; ^3^Department of Neurosurgery, 904th Hospital of Joint Logistic Support Force of PLA, Wuxi Clinical College of Anhui Medical University, Wuxi, China

**Keywords:** cell death, SAH, necrosis, apoptosis, ferroptosis, autophagy, pyroptosis

## Abstract

Subarachnoid haemorrhage (SAH) is a common cerebrovascular disease with high disability and mortality rates worldwide. The pathophysiological mechanisms involved in an aneurysm rupture in SAH are complex and can be divided into early brain injury and delayed brain injury. The initial mechanical insult results in brain tissue and vascular disruption with hemorrhages and neuronal necrosis. Following this, the secondary injury results in diffused cerebral damage in the peri-core area. However, the molecular mechanisms of neuronal death following an aneurysmal SAH are complex and currently unclear. Furthermore, multiple cell death pathways are stimulated during the pathogenesis of brain damage. Notably, particular attention should be devoted to necrosis, apoptosis, autophagy, necroptosis, pyroptosis and ferroptosis. Thus, this review discussed the mechanism of neuronal death and its influence on brain injury after SAH.

## Introduction

Subarachnoid haemorrhage (SAH) is a common cerebrovascular disease, accounting for approximately 5% of all strokes. According to the latest report by the European Stroke Association, the incidence of SAH is reported to be more than 9/100,000 (Feigin et al., 2009; GBD 2019 Stroke Collaborators, 2021). The incidences of SAH in Asia and Iceland rank the highest, with Japan accounting for approximately 22.7/100,000 cases. Moreover, more than 85% of all SAH cases are caused by intracranial aneurysms, leading to high mortality and disability rates (Steiner et al., 2013). A meta-analysis reported that the short-term mortality of SAH is as high as 8.3–66.7%, the mean median of direct death before admission was 8.3% and the overall favorable prognosis rate was 36–55% (Nieuwkamp et al., 2009). The majority of SAH survivors usually have long-term cognitive dysfunction, affective disorders, loss of hearing and smell and decreased quality of life after surgery (Rinkel and Algra, 2011). Additionally, >60% of SAH survivors have memory problems, >75% have language problems (Al-Khindi et al., 2010) and almost 95% of patients with SAH have at least one cognitive or affective disorder (Passier et al., 2010). The treatment for patients with SAH is lengthy and expensive, which places a serious burden on the national economy. Since the beginning of the 21st century, the average hospitalization cost of each patient with a SAH aneurysm in the first year in the United States exceeded $65,000 (Qureshi et al., 2007) while those in the United Kingdom paid £510 million a year (Rivero-Arias et al., 2010).

The pathophysiological processes and mechanisms involved in aneurysm rupture in SAH are complex. Nonetheless, a previous study divided the pathological process into two stages: early brain injury (EBI) and delayed brain injury (DBI) (Chen S. et al., 2014). Additionally, various animal and cell experimental studies on SAH report that a large number of red blood cells enter the subarachnoid space, brain parenchyma or ventricle and their subsequent rapid lysis produce a large amount of hemoglobin and corresponding decomposition products (Macdonald, 2014; Lawton and Vates, 2017). These factors exert various neurotoxic effects and can subsequently induce secondary brain injury after SAH (Bulters et al., 2018; Chen et al., 2019c, 2021). Therefore, EBI is considered the most important cause of acute brain damage and neurological dysfunction in patients with SAH. Currently, it is gaining increasing attention as a research hotspot. Neuronal death is considered a major cause of EBI (Macdonald and Schweizer, 2017). Hence, a reduction in the number of neuronal deaths could improve nerve dysfunction and eventually improve the prognosis of patients (Dong et al., 2016; Ding et al., 2022). Thus, further study on the mechanism of EBI and neuronal death is vitally important. In the present review, we discuss the mechanism of neuronal death after SAH and its influence on brain injury (Table 1).

**TABLE 1:** Neuronal death pathways and associated morphological and biochemical hallmark features.

Neuronal death	Morphological features and key biochemical pathway components	References
Neuronal necrosis	Plasma membrane rupture, swelling of neuronal organelle, deformation, nucleus changes, DNA dissolution, intracellular calcium overload, sodium ion influx, potassium ion outflow plasma concentration changes, and overspending membrane aquaporin, lack of inter-nucleosomal DNA fragmentation, depletion of ATP, involvement of calpains and cathepsins, release of DAMPs (and in infected cells also PAMPs)	Kroemer et al., 2009; Friedrich et al., 2012
Neuronal apoptosis	Nuclear fragmentation, plasma membrane blebbing, cell shrinkage (pyknosis), formation of apoptotic bodies and phagocytosis by neighboring cells. Pro-apoptotic BCL-2 family members, P53 and caspase activation, cleavage of hundreds of caspase substrates (ICAD, PARP), PS exposure, ΔΨm dissipation, MOMP and ROS production	Chen J. H. et al., 2016; Chen et al., 2018b; Chen et al., 2019c
Neuronal pyroptosis	Rupture of the plasma membrane and lack of cell swelling. Inflammatory induced activation of the initiator caspases, caspase-1, -4, -5 and -11, and consequent activation of the effector caspases, caspase-3 and -1. Release of bio-active IL-1β and IL-18 and proteolytic activation of GSDMD	Bergsbaken et al., 2009; Aglietti et al., 2016; Liu et al., 2016; Vande Walle and Lamkanfi, 2016
Neuronal autophagy	Accumulation of autophagic vacuoles, autophagosome, vacuolisation of the cytoplasm, no chromatin condensation. Atg family of gene encoded proteins, LC3-I to LC3-II conversion and cleavage of p62	Ho et al., 2018
Neuronal necroptosis	Cytoplasmic swelling, loss of plasma membrane integrity, swelling of cytoplasmic organelles. RIPK1, RIPK3, MLKL, phosphorylation and ubiquitination of RIPK1, formation of the necrosome complex in the cytosol, phosphorylation and activation of MLKL, the effector of caspases, ROS production and release of DAMPs	Holler et al., 2000; He et al., 2009; Galluzzi et al., 2017
Neuronal ferroptosis	Smaller mitochondria with decreased cristae, disappearance of mitochondrial cristae, thickening of the lipid bilayer membrane, metabolic dysfunction of iron ions, depletion of glutathione (GSH), accumulation of iron-dependent lipid overreactive oxygen species (ROS), inhibited activity, or decreased levels of glutathione peroxidase 4 (GPX4)	Dixon et al., 2012; Zille et al., 2017; Chen et al., 2020c

## Neuronal necrosis

**FIGURE 1:**
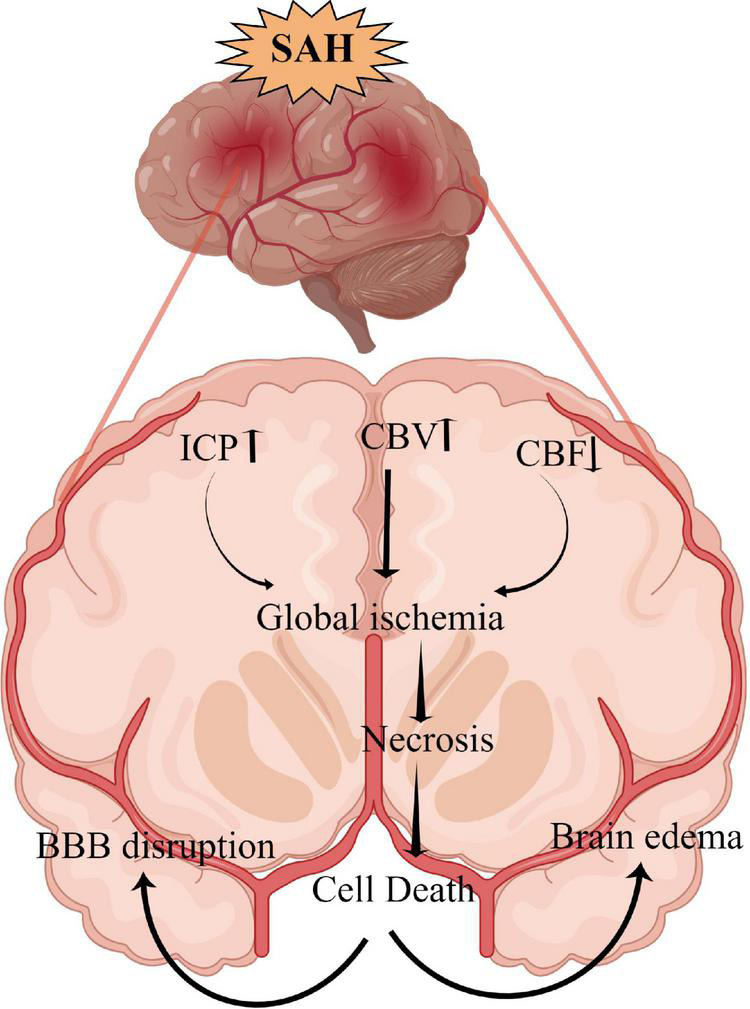
The overall scheme of neuronal necrosis from subarachnoid hemorrhage to early brain injury (EBI).

Neuronal necrosis is the death of neurons induced by extreme, chemical or severe pathological factors and is a passive pathological process that does not consume energy (Kroemer et al., 2009). The morphology of necrotic neurons exhibits increased neuronal membrane permeability, neuronal organelle oedema and deformation, nuclear changes, DNA dissolution, intracellular calcium overload, sodium ion influx, potassium ion outflow, plasma concentration changes (Friedrich et al., 2012). Furthermore, degraded lysosomes release various cellular autolysins that eventually destroy the plasma membrane and cause cell death (Kroemer et al., 2009). This pathological process is also an acute passive neuronal death process that is observed after SAH. The specific mechanism and molecular process of neuronal necrosis after SAH are scarce, to the best of our knowledge, as it is a passive, unregulated and irreversible pathological process (Zhu et al., 2012). Friedrich et al. (2012) reported that the activation of endothelial and parenchymal cell apoptosis and neuronal necrosis occurred within 10 min after SAH and is associated with poor outcomes. Thus, the prevention of apoptosis and necrosis could potentially improve impaired functions of the circumventricular organs after SAH (Edebali et al., 2014). However, neuronal necrosis is the most common form of neuronal death in EBI after SAH and is also the main cause of early death, coma and severe neurological dysfunction in patients after SAH (Friedrich et al., 2012). Although neuronal necrosis is a passive and unregulated process, early clinical intervention against its inducing factors can significantly reduce its occurrence (Zhu et al., 2012). Therefore, identifying and inhibiting the inducers of neuronal necrosis is key to regulating this process, which could also be a new research direction for neuronal necrosis after SAH. Additionally, Figure 1 briefly summarizes the clinical causes of neuronal necrosis after SAH.

### Intracranial hypertension

SAH can lead to extensive hemorrhage, including intraventricular hematoma and intracerebral hematoma, and the mass effect directly leads to increased intracranial pressure (ICP) (Macdonald and Schweizer, 2017). Heuer et al. (2004) reported that intracranial hypertension (>20 mmHg) occurs in more than 50% of patients with SAH, with rates as high as 60–70% in poor-grade SAH. Similarly, Zoerle et al. (2015) reported that >80% of patients with SAH had at least one episode of intracranial hypertension following SAH and 36% had an average ICP of >20 mmHg during hospitalization. Increased ICP and complications, such as cerebral ischemia and brain oedema, can directly induce neuronal necrosis, affecting the final prognosis of patients (Said et al., 2021; Narotam et al., 2022). Although the relationship between ICP and prognosis remains unclear, the definite presence of intracranial hypertension after SAH increases the mortality rates and leads to poor outcomes. Moreover, intracranial hypertension and its etiology (hydrocephalus, cerebral oedema, intracerebral hematoma and intraventricular hematoma.) can be used as an independent predictor of poor patient outcome (Claassen et al., 2002; Heuer et al., 2004; Zoerle et al., 2015; Wan et al., 2016).

Currently, there are no recommended guidelines for the treatment of intracranial hypertension after SAH (Svedung Wettervik et al., 2022). From our experience, early-time intraventricular ICP monitoring is a very important therapeutic strategy to relieve ICP in patients with SAH. It is also essential to monitor ICP and cerebral perfusion pressure to avoid severe cerebral ischemia, which can be avoided by maintaining appropriate perfusion pressure regardless of vasospasm (Chen et al., 2018a). Additionally, intraventricular ICP monitoring can aid in properly draining cerebrospinal fluid, reducing ICP, improving obstructive hydrocephalus, draining blood-cerebrospinal fluid to improve vasospasm and reducing toxic brain injury (He et al., 2015; Chen J. et al., 2016). However, not all patients with SAH require ventricular ICP monitoring but it can be considered for poor-grade patients with SAH as the invasive procedure could increase the risk of additional injury and intracranial infection (Chen et al., 2018a). Olson et al. (2013) reported that continuous cerebrospinal fluid drainage and intermittent ICP monitoring increased the incidence of intracranial infection, cerebrospinal fluid leakage and bleeding (clinicaltrials.gov identifier NCT01169454) in a randomized controlled study of 60 patients. Hence, it is necessary to adopt individualized precision therapy to control intracranial hypertension, thereby reducing neuronal necrosis and ameliorating EBI.

### Cerebral ischemia

Cerebral ischemia is a common complication after SAH, with >30% of patients experiencing delayed cerebral ischemia (Stienen et al., 2014). Decreased cerebral hypoperfusion leads to cerebral ischemia and cerebral infarction, ultimately inducing severe neurological dysfunction, brain injury and even death (Güresir et al., 2022; Hostettler et al., 2022). Cerebral ischemia is caused by various factors, including cerebral vasospasm, micro thrombosis, blood-brain barrier destruction, inflammatory reactions and cerebral vascular self-regulation dysfunction (Macdonald, 2014). After SAH, a large amount of hemoglobin enters the subarachnoid space to directly stimulate intracranial blood vessels, thereby activating calcium channels in vascular smooth muscle cells and resulting in smooth muscle contraction and vascular spasm (Pluta, 2005). Endothelin-1 (ET-1) is produced in large quantities by astrocytes and white blood cells during inflammation or cerebral ischemia and has a strong vasoconstriction function (Chen et al., 2018b; Croci et al., 2019). Previous studies have demonstrated that many molecular signaling pathways, such as p38/MAPK (Huang et al., 2017), RhoA (González-Montelongo et al., 2018) and Nrf2-ARE (Zhang et al., 2010; Zhao et al., 2016), are involved in the development of cerebrovascular spasms (CVS) after SAH. Therefore, early diagnosis and treatment of CVS and cerebral ischemia could improve the prognosis of patients. Regrettably, a large number of drugs are effective in basic research but ineffective in clinical trials. For example, clazosentan has been considered for the targeted treatment of CVS; however, a Phase 3 clinical trial found that while it improved CVS, it did not improve the long-term outcomes for patients with SAH (Macdonald et al., 2011). Previous studies report that statins can protect against CVS and EBI after SAH (Chen J. H. et al., 2016; Chen et al., 2018b, 2020a, 2021); however, another study indicates that statins together with nimodipine can reduce CVS and cerebral infarction but have no benefits on 6 months of clinical outcome or 30-day all-cause mortality (Chen et al., 2020b). Additionally, magnesium is gaining widespread attention; however, it has also proven to be ineffective in Phase 2 randomized controlled trials (Mees et al., 2012). Recent clinical studies on cilostazol have demonstrated that it can significantly reduce the incidence of cerebral infarction, delay ischemia after SAH (Sugimoto et al., 2018), reduce the occurrence of secondary stroke and improve the prognosis of patients (Shinohara et al., 2010; Toyoda et al., 2019). However, its effect on ameliorating CVS and micro thrombosis after SAH and improving prognosis require further study using a large sample, multicentre, randomized controlled trial. Additionally, the relationship between CVS and neuronal necrosis after SAH remains unclear and requires further exploration.

### Brain oedema

Brain oedema after SAH can be divided into three types: vasogenic cerebral oedema, cytotoxic cerebral oedema, and mixed cerebral oedema (Nag et al., 2009). Vasogenic cerebral oedema is the most common type of brain oedema in the clinical setting, whereas cytotoxic cerebral oedema is caused by increased permeability due to cell membrane disruption and swelling (Kaufmann and Cardoso, 1992). Notably, after SAH, different types of cerebral oedema mainly occur at different periods and stages (Nag et al., 2009). The expression of matrix metalloproteinase-9 is increased after SAH, which leads to the destruction of tight junction proteins between the vascular endothelium and subsequently results in the increased permeability of the blood-brain barrier and aggravated brain oedema (Lee et al., 2007; Sun X. G. et al., 2019). The activation of aquaporin-4 (AQP4) is also an important mechanism affecting brain oedema, wherein water molecules enter brain tissues in large quantities through the activated AQP4 and then aggravate brain oedema and neuronal necrosis in cytotoxic cerebral oedema (Huang et al., 2014; Chen J. H. et al., 2016). In vasogenic cerebral oedema, AQP4 plays an important role in improving oedema, which was confirmed by the significantly reduced rate of resolution of cerebral oedema in AQP4 knockout cells (Papadopoulos et al., 2004; Tourdias et al., 2011). Progressive cerebral oedema leads to increased ICP and malignant intracranial hypertension and subsequently to cerebral ischemia and neuronal necrosis (Bjerring et al., 2011; Halstead and Geocadin, 2019). Therefore, the early management of cerebral oedema after SAH is vitally important in improving the prognosis of patients with SAH, especially those with poor-grade aneurysmal SAH. Powell et al. (1976) also reported that the extent of swelling was significantly decreased with a dehydrating agent (mannitol) treatment and osmolality elevation decreased the extent of eventual myocardial necrosis. Additionally, dehydrating agents also improve brain tissue oxygenation by limiting astrocyte swelling and restoring capillary perfusion, thereby alleviating early brain injury (Halstead and Geocadin, 2019). Hence, exploring the treatment of neuronal necrosis with dehydrating drugs and AQP4-based targeted drugs has therapeutic potential.

## Neuronal apoptosis

**FIGURE 2:**
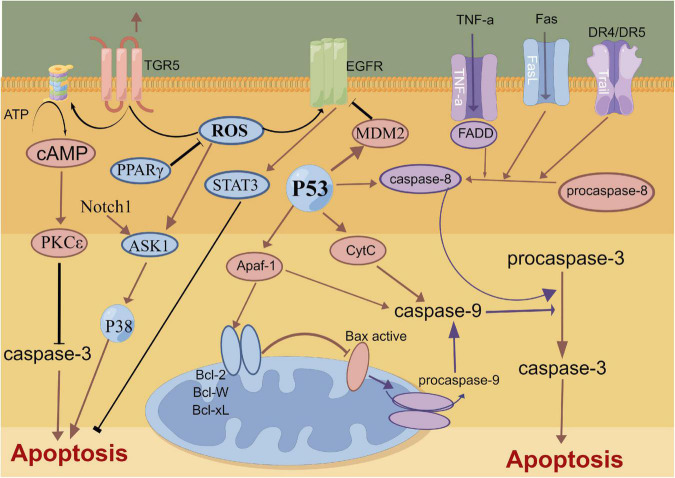
Molecular mechanisms of apoptosis from subarachnoid hemorrhage to early brain injury (EBI).

The mechanical compression of hematoma, toxic damage of hemoglobin and oxidative stress lead to the extensive apoptosis of cortical neurons and hippocampal neurons after SAH (Chen J. H. et al., 2016; Chen et al., 2018b, 2019c). The complex process of neuronal apoptosis involves exogenous death receptor pathways, P53-mediated apoptosis pathways, caspase-dependent and -non-dependent pathways and endogenous mitochondrial pathways (Gao et al., 2000; Sendoel et al., 2010). The BcL-2 gene family promotes the release of cytochrome C from the mitochondrial membrane and its binding with apoptosis protease activating factor 1 (Apaf-1) to regulate mitochondrial membrane permeability (Hasegawa et al., 2011; Jaafaru et al., 2019). The c/Apaf-1 complex further regulates the activation of the apoptosis factor Caspase-3 and induces neuronal apoptosis (Hasegawa et al., 2011). Recent study report that a novel membrane-bound bile acid receptor (TGR5) is involved in the process of oxidative stress and apoptosis, wherein TGR5 and its agonists inhibit neuronal apoptosis and improve neural function via the cAMP/PKCε/ALDH2 pathway in a rat SAH model (Zuo et al., 2019). Furthermore, protein-coupled receptor 30 (GPR30) and its agonist G1 are also involved in neuronal apoptosis after SAH, which can inhibit neuronal apoptosis through the src/EGFR/STAT3 signaling pathway (Peng et al., 2019). A previous study showed that statins can alleviate neuronal apoptosis and EBI by inhibiting caspase-3 after SAH (Chen J. H. et al., 2016). Additionally, Netrin-1 can activate PPARγ and Bcl-2 expressions, inhibit the proapoptotic factors Bax and NF-κB and ameliorate EBI after SAH (Chen et al., 2019c). Similarly, Gao et al. also reported that the early overexpression of PDK4 after SAH has the potential to attenuate neuronal apoptosis by reducing oxidative stress via the ROS/ASK1/P38 pathway (Gao X. et al., 2022). A recent study also demonstrated that early fluoxetine administration can protect against apoptosis by regulating the Notch1/ASK1/p38 MAPK signaling pathway, thereby ameliorating EBI after SAH (Liu M. et al., 2022). Neuronal apoptosis caused by SAH is widely and thoroughly studied, involving many signaling pathways and molecular mechanisms. Future studies need to assess and identify specific molecular mechanisms that can be regulated in the induction of apoptosis, thereby contributing to the development of clinically viable treatment options (Figure 2).

## Neuronal pyroptosis

**FIGURE 3:**
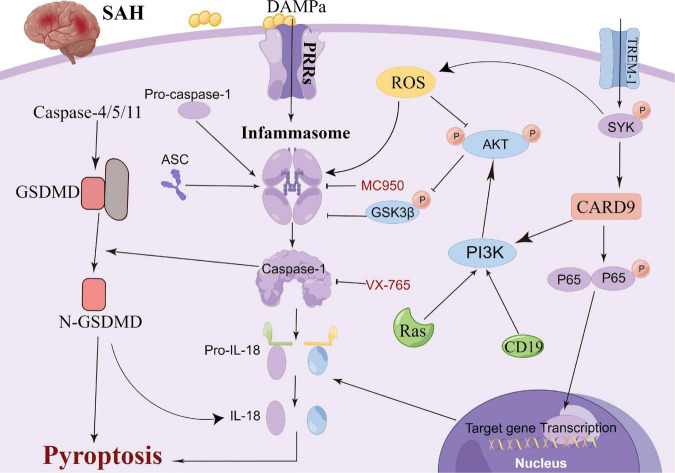
Molecular mechanisms of pyroptosis from subarachnoid hemorrhage to early brain injury (EBI).

Pyroptosis, a newly reported cell death mechanism, is a form of programmed neuronal cell death initiated by Caspase-1 and an important cause of neurological damage (Bergsbaken et al., 2009). Pyroptosis, which was first proposed in 2001, differs from apoptosis with respect to its mechanism, which is associated with inflammation (Aglietti et al., 2016; Liu et al., 2016; Vande Walle and Lamkanfi, 2016). Pyroptosis is a programmed cell death mediated by GSDMD (gasdermin protein family, including Gasdermin A, Gasdermin B, Gasdermin C, DFNA5 and DFNB59), with the inflammasome playing an important role in the pyroptosis process (Kayagaki et al., 2015). Gasdermin, an important element of cell pyrosis, is also widely distributed in the central nervous system (Bergsbaken et al., 2009). Hence, neuronal pyrosis plays an important role in the development of central nervous system-related diseases and is an important mechanism of brain injury (Ito et al., 2015; Van Rossom et al., 2015; An et al., 2019; Kuwar et al., 2019; She et al., 2019; Sun Y. B. et al., 2019). In the classic Caspase-1-induced cell pyroptosis process, inflammasomes (NLRP1, NLRP3, NLRC4 and AIM2) sense the danger signal and induce the connector molecule ASC to activate Caspase-1, thus cleaving GSDMD (Swanson et al., 2019; Chen et al., 2022). Subsequently, cellular pores are formed on the cell membrane, which results in water and ion influx and ultimately cell swelling and death (Shi et al., 2015; Broz and Dixit, 2016). Non-classical pathway-induced cell pyroptosis is activated by the oligomerization of the non-classical inflammasome (in mice, a complex of Caspase-11 precursors and lipopolysaccharides), which binds the intracellular Caspase-11 precursors to lipopolysaccharides (Abu Khweek and Amer, 2020). Following this, GSDMD is cleaved by activation of the Caspase-4/5/11 pathway, which is independent of Caspase-1, to induce cell pyroptosis (Kayagaki et al., 2013; Diamond et al., 2015). Moreover, in animal ischemic stroke experiments, microglial TREM-1 induced the activation of spleen tyrosine kinase (SYK), which increased the level of the N-terminal fragment of GSDMD (Liang et al., 2020). Thereafter, pores were formed in the microglia to promote the release of intracellular inflammatory factors, leading to the pyroptosis of the microglia. Additionally, microglial TREM-1 receptors bind to SYK and induce neuroinflammatory damage after stroke (Xu et al., 2019). Kuwar et al. (2019) also reported that inflammasome inhibitors could significantly inhibit the pyroptosis of neurons and improve brain injury after traumatic brain injury. Additionally, statins have been reported to improve neurological outcomes and reduce neuronal death against neural pyroptosis and neuroinflammation in an animal SAH model (Chen et al., 2021). Gu et al. (2022) demonstrated that the activation of RKIP could attenuate neuronal pyroptosis and brain injury after ICH through the ASC/Caspase-1/GSDMD pathway. Similarly, the inhibition of the pyroptosis of astrocytes and microglia in the central nervous system can also indirectly inhibit the pyroptosis of neurons, thereby protecting neurons and alleviating brain injury (Chavez et al., 2021; Kerr et al., 2022). Collectively, these studies highlight the sophisticated interplay between pyroptosis-related molecules and inflammasome components that could be leveraged to prevent hyperinflammation after SAH (Figure 3). Thus, pyroptosis can be a new research direction in the exploration of the mechanism of SAH.

## Neuronal autophagy

**FIGURE 4:**
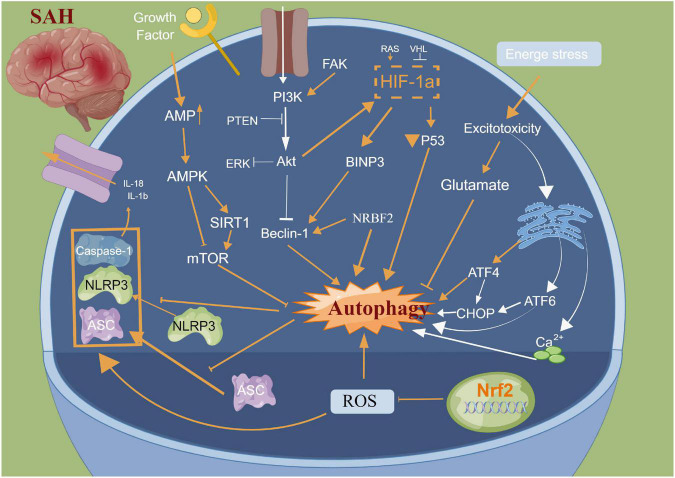
Molecular mechanisms of autophagy from subarachnoid hemorrhage to early brain injury (EBI).

Autophagy is a cellular repair process that stabilizes the intracellular environment via the lysosomal degradation of selected cytoplasmic components and programmed death of old, dysfunctional or unnecessary cytoplasmic entities (Ho et al., 2018). Autophagy is tightly regulated and involves multiple molecular mechanisms; autophagy plays an important role after SAH (Chen J. et al., 2014; Li et al., 2017; Ho et al., 2018; Sun C. et al., 2019; Sun C. M. et al., 2019). The main morphological changes have been observed in the autophagosomes and autophagolysosomes under an electron microscope (Harris and Rubinsztein, 2012; Chen J. et al., 2014; Li et al., 2017; Ho et al., 2018; Sun C. et al., 2019; Sun C. M. et al., 2019). Lee et al. (2009) reported that neuronal autophagy was significantly activated in the early injury stage of SAH, and different modes of autophagy activation in different parts of SAH indicated different injury mechanisms. Wang et al. (2012) reported that a small amount of autophagy exists in normal brain neurons; however, it is activated in large quantities after SAH. Autophagy can improve cerebral oedema, protect the blood-brain barrier, reduce cortical neuronal apoptosis and improve clinical prognosis after intervention with rapamycin (RAP) and 3-methyladenine (3-MA) (Hu X. et al., 2022; Pang et al., 2022). Lu et al. reported that melatonin enhances autophagy in brain vessel endothelial cells, then protect blood-brain barrier integrity. In the animal CVS model after SAH, the autophagy pathway was activated in the basilar artery wall after SAH, and CysC-induced autophagy played a beneficial role in preventing SAH-induced CVS (Liu et al., 2013). Additionally, many autophagy-related drugs have achieved good neuroprotective effects in basic research (Li et al., 2017; Chen et al., 2020a; Du et al., 2021; Chang et al., 2022). Sun C. et al. (2019) reported that osteopontin enhanced the autophagy response after SAH via the activation of the FAK-ERK signaling pathway, thereby improving long-term neurological function. Furthermore, Li and Han (2018) confirmed using *in vivo* and *in vitro* models that resveratrol increased the LC3-II/I ratio, AMPK phosphorylation and SIRT1 expression, thereby activating autophagy and reducing the release of inflammatory factors and neuronal apoptosis. Li et al. (2017) reported that fluoxetine significantly decreased the expression of NLRP3 and Caspase-1 and upregulated the expression of Beclin-1, which was reversed by the autophagy inhibitor 3-MA. Additionally, fluoxetine can enhance autophagy by inhibiting the activation of the NLRP3 inflammasome, thus playing a neuroprotective role, which could be attributed to the interaction between autophagy and pyroptosis (Li et al., 2017). Arachidonyl-2-chloroethylamide (ACEA), a highly selective CB1R agonist, was also reported to alleviate oxidative stress and neurological dysfunction by promoting mitophagy after SAH via the CB1R/Nrf1/PINK1 signaling pathway (Liu B. et al., 2022). Similarly, Zeng et al. (2021) demonstrated that the autophagy protein NRBF2 alleviated endoplasmic reticulum stress-associated neuroinflammation and oxidative stress by promoting autophagosome maturation, which occurs through interactions with Rab7 after SAH, and ultimately improving EBI. Hence, autophagy plays an important role in neuronal death, blood?brain barrier and CVS after SAH. Further studies focusing on elucidating the structural basis of the autophagy interaction for pyroptosis induction and developing autophagy-related drugs need to be explored in clinical trials (Figure 4).

## Neuronal necroptosis

**FIGURE 5:**
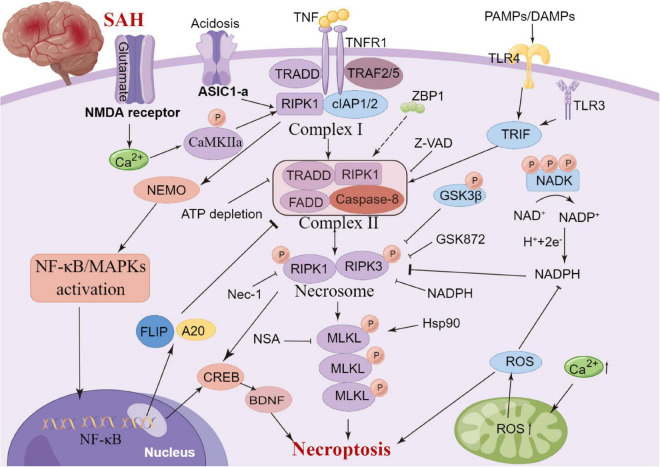
Molecular mechanisms of necroptosis from subarachnoid hemorrhage to early brain injury (EBI).

Necroptosis is a regulated form of cell death that relies heavily on receptor interactions of serine-threonine kinase 3 (RIPK3) and the mixed lineage kinase domain (MLKL) and usually presents with morphological characteristics of necrosis (Galluzzi et al., 2017). Unlike apoptosis, necroptosis is a caspase-independent mode of cell death. It is now known that serine/threonine kinases RIPK1, RIPK3 and MLKL are critical for necroptosis induced by the tumour necrosis factor (TNF) (Holler et al., 2000; He et al., 2009)superfamily, TLR3 or TLR4 and interferon receptors (Kaiser et al., 2013). TNF-α-induced necroptosis is the most characteristic signal transduction pathway. TNF-α homotrimer binds to TNF-1 to form a membrane signaling complex-1, which includes TRADD, RIPK1, TRAF2 and cIAP1/cIAP2, and subsequently activates NF-κB and MAP kinases (Chen and Goeddel, 2002). The polyubiquitination of RIPK1 by CIAP1/CIAP2 and LUBC is essential for the activation of the NF-κB signaling pathway (Gerlach et al., 2011). RIPK1 and RIPK3 in the necrosome are phosphorylated by their amino-terminal kinase domains, resulting in cell death through a signaling cascade (He et al., 2009). A previous review summarized that necroptosis is widely associated with various cardiovascular diseases, nerve regeneration diseases, kidney injury and other diseases (Galluzzi et al., 2017). Degterev et al. (2005) observed that necroptosis leads to delayed ischemic brain injury in mice through a mechanism that is different from apoptosis, thereby identifying a previously undescribed basic cell death pathway and providing a new therapeutic target for stroke. Chen et al. (2019a) reported that anti-TNF-α treatment can significantly improve endothelial cell necroptosis and blood-brain barrier damage and further improve nerve function after stroke. In a SAH experimental study, celastrol administration decreased the expression levels of necroptosis-related proteins RIPK3 and MLKL, which exhibited neuroprotective effects on EBI (Xu et al., 2021). Similarly, necrostatin-1, a small-molecule inhibitor of necroptosis, can potentially prevent SAH-induced necroptosis by suppressing the activity of the RIPK3/MLKL signaling pathway (Chen et al., 2019b). Therefore, the inhibition of necroptosis can significantly improve neural function, and its mechanism could be related to the phosphorylation of RIPK1 and RIPK3 (Shen et al., 2017; Yuan et al., 2019). Yang et al. (2018) reported that the specific and potent inhibitor of necroptosis Necrostatin-1 (Nec-1) mitigated SAH-induced synaptic impairments in the hippocampus by inhibiting necroptosis via the CREB-BDNF pathway. Additionally, Nec-1 has been reported to prevent CVS in animals models (Sahin et al., 2021). However, the clinical efficacy of drugs targeting the mechanism of necroptosis remains unclear owing to a lack of relevant clinical studies. Further studies on anti-necroptosis-related drugs that can be used in clinical settings against brain injury after SAH are essential. Additionally, clinical trials should be performed to evaluate the safety and clinical efficacy of anti-necroptosis-related drugs, such as Nec-1 (Figure 5).

## Neuronal ferroptosis

**FIGURE 6:**
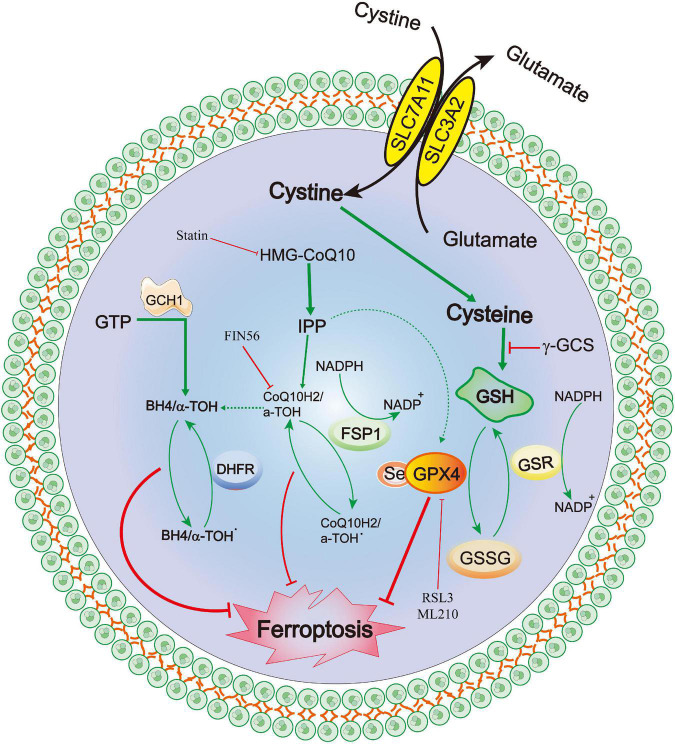
Molecular mechanisms of ferroptosis from subarachnoid hemorrhage to early brain injury (EBI).

Recently, a newly identified cell death mechanism called ferroptosis has been widely explored. It is a non-apoptotic form of iron-dependent programmed cell death and differs from traditional cell death processes, such as apoptosis and autophagy (Dixon et al., 2012; Chen et al., 2020c). Moreover, ferroptosis cannot be completely inhibited by necroptosis/apoptosis/autophagy-related inhibitors and intracellular calcium overload (Zille et al., 2017). The classical morphological characteristics of ferroptosis are the disappearance of the mitochondrial cristae, significantly narrowed mitochondria, thickening of the lipid bilayer membrane and reduced cell connections that lead to cell separation (Chen et al., 2020c). Furthermore, the biological characteristics mainly include the metabolic dysfunction of iron ions, depletion of glutathione (GSH), accumulation of iron-dependent lipid reactive oxygen species (ROS) and decreased levels or inhibition of glutathione peroxidase 4 (GPX4) (Chen et al., 2020c). The mechanisms of ferroptosis are complex and involve various cell signaling pathways (Chen et al., 2020c, 2021). Recent studies have also confirmed that ferroptosis is widely observed in diseases of the central nervous system, including cerebral ischemia/reperfusion (Tuo et al., 2022), SAH (Gao S. et al., 2022), ICH (Dharmalingam et al., 2020) and traumatic brain injury (Rui et al., 2021). The GPX4–GSH–cysteine system, FSP1–CoQ10–NAD (P)H system and GCH1-BH4-DHFR system are the primary anti-ferroptosis systems. A better understanding of three primary anti-ferroptosis systems aids in developing better therapeutic strategies for patients with brain injury after SAH (Figure 6).

### GPX4–GSH–cysteine system

Ferroptosis was first reported to induce iron-dependent cell death via the small molecules erastin and 1S,3R-RSL3 (RSL3), a classical feature of ferroptosis is that it is independent of Caspase activation after erastin and RSL3 treatment (Dixon et al., 2012; Wang et al., 2016). Erastin targets the Xc- system (comprising SLC3A2 and SLC7A11), which transports cysteine into cells and glutamate out of cells, Cysteine is required for the synthesis of GSH, an important antioxidant that protects cells from oxidative damage (Dixon et al., 2012). Furthermore, erastin inhibits intracellular cysteine, leading to a significant decrease in intracellular GSH levels, ROS levels and oxidative damage (Dixon et al., 2012). GPX4 is the core of ferroptosis and requires GSH as a substrate for lipid repair (Yang et al., 2014). Reduced cysteine levels lead to the depletion of GSH levels and reduction of GPX4 levels and activity, resulting in the accumulation of unrepaired lipid peroxides and ferrous ions (Friedmann Angeli et al., 2014; Stockwell et al., 2017). GSH is a very important component of Fe^2+^ in unstable iron pools, it binds to Fe^2+^ to prevent lipid peroxidation, directly inhibiting GSH biosynthesis and triggering ferroptosis (Hider and Kong, 2011). Additionally, GPX4 can reduce the level of lipid hydrogen peroxide via GSH and convert it to ethanol or free hydrogen peroxide (Stockwell et al., 2017; Gaschler et al., 2018; Forcina and Dixon, 2019), which can be inhibited by RSL-3 (Dixon et al., 2012; Yang et al., 2014), and FINO2 (Gaschler et al., 2018). Conversely, selenium is a key regulator of GPX4 activity (Ingold et al., 2015, 2018; Friedmann Angeli and Conrad, 2018; Alim et al., 2019) and its supplementation after stroke can effectively inhibit ferroptosis and cell death after haemorrhagic or ischemic stroke (Alim et al., 2019).

### FSP1–CoQ10–NAD(P)H

Recently, a novel apoptosis-inducing factor mitochondria-associated 2 (AIFM2) gene, also known as AIF homologous mitochondria-related death inducer, has been reported as an anti-ferroptosis gene (Bersuker et al., 2019; Doll et al., 2019). Doll et al. (2019) renamed AIFM2 as FSP1 (ferroptosis suppressor protein 1, FSP1). It has been reported to supplement the anti-iron death effect in the absence of GPX4 by using NAD(P)H to catalyze CoQ10 regeneration and then alleviate lipid peroxidation. Shimada et al. also confirmed that NADPH levels could be used as an important marker of the sensitivity of many cancers cell lines to ferroptosis (Shimada et al., 2016). The FSP1-CoQ10-NAD(P)H pathway is a parallel anti-ferroptosis system independent of the cystine-GSH-GPX4 system that inhibits lipid peroxidation and anti-ferroptosis effects, FSP1 has a dual regulatory role: inducing apoptosis and preventing ferroptosis (Wu et al., 2002; Bersuker et al., 2019; Dar et al., 2021; Tonnus et al., 2021). Furthemore, the C-terminal fragment of FSP1 located in the outer membrane of the mitochondria induced apoptosis while the two N-terminal fragments located in the nucleus cannot induce apoptosis (Wu et al., 2002). Kaku et al. (2015) reported that the translocation of endogenous FSP1 into the nucleus is a necessary step in the apoptosis mechanism. Additionally, FSP1 is an oxidoreductase of CoQ10 (CoQ10), and the nutmeg acylation of the N-terminal of FSP1 is a lipid modification that promotes FSP1 translocation to the plasma membrane, thereby mediating NADH-dependent CoQ reduction on the plasma membrane to inhibit CoQ10 activity (Wu et al., 2002; Bersuker et al., 2019; Dar et al., 2021; Drijvers et al., 2021; Song Z. et al., 2021; Tonnus et al., 2021). Notably, FSP1 still plays an anti-ferroptosis role even after GPX4 knockdown. Therefore, FSP1-CoQ10-NAD(P)H is an independent pathway that cooperates with the cystine-GSH-GPX4 system to inhibit ferroptosis. In a Parkinson’s disease model, Song L. M. et al. (2021) demonstrated that inhibiting the upregulation of long-chain acyl-CoA synthase 4 (ACSL4) and downregulation of ferroptosis inhibitor 1 (FSP1) can effectively prevent MPTP-induced ferroptosis, thereby identifying a promising therapeutic target for Parkinson’s disease.

### GCH1-BH4-DHFR

Exogenous dopamine or melatonin has been shown to inhibit ferroptosis induced by heme (Song L. M. et al., 2021). Tetrahydrobiopterin (BH4) is a natural nutrient that is involved in the biosynthesis of neurotransmitters, for example, 5-hydroxytryptamine, dopamine, adrenaline and melatonin can be used as cofactors of various enzymes, such as tryptophan hydroxylase, nitric oxide (NO) synthase and glycerol ether monooxygenase (Werner et al., 2011). BH4 plays a redox role in the formation of NO, which is catalyzed by L-arginine and NADPH (Hu Q. et al., 2022). The oxidation of BH4 to BH2 leads to the uncoupling of NOS to form O2^⋅−^, which reacts rapidly with NO to form peroxynitrite and further decouple NOS (Song L. M. et al., 2021). Deng et al. (2020) also confirmed that the activation of the NO pathway was associated with tissue damage related to ferroptosis. Additionally, Soula et al. (2020) indicated that BH4 synthesis is a necessary molecule required to protect cells from death. Moreover, the expression of GTP cyclohydrolase 1 (GCH1) determines the effectiveness of BH4 and its dependence on GPX4 inhibition (Kraft et al., 2020; Cronin et al., 2022). BH4 prevents the accumulation of lipid peroxides independently of its cofactor and is an effective free radical trapping agent and antioxidant in the lipid membrane, Dihydrofolate reductase catalyzes BH4 regeneration, methotrexate and GPX4 have been reported to synergistically inhibit ferroptosis (Hu Q. et al., 2022). Additionally, GCH1-mediated BH4 production prevents ferroptosis by inhibiting lipid peroxidation (Kraft et al., 2020; Hu Q. et al., 2022). In the hypertension model reported by Wang et al. (2021), the antihypertensive effect of L-phenylalanine could be mediated by enhancing BH4 biosynthesis and reducing superoxide levels produced by NO synthase, thereby reducing ROS and improving NO levels and protecting blood vessels and renal function. Hence, the GCH1-BH4 signaling pathway, as an endogenous antioxidant pathway, inhibits iron death through a mechanism independent of the GPX4/GSH system (Kuang et al., 2020; Wei et al., 2020).

## Conclusions and prospects

The pathological processes and mechanisms of SAH rupture are extremely complex. Regardless of the main mechanism, blood entering the subarachnoid space or brain tissue is considered the inducer. Furthermore, the subsequent red blood cell lysis products release various toxic substances and lead to the death of neurons, ultimately leading to secondary brain injury (Bulters et al., 2018). Increasing basic studies have also demonstrated that ferroptosis-specific inhibitors, including liproxstatin-1 and ferrostatin-1, can decrease lipid peroxidation and neuronal ferroptosis after SAH *in vivo* and *in vitro* by regulating GPX4 and ACSL4 (Cao et al., 2021; Li et al., 2021; Qu et al., 2021). The present review expounds on the roles and related mechanisms of neuron necrosis, apoptosis, pyroptosis, necroptosis, autophagy and ferroptosis in brain injury after SAH. These mechanisms are interwoven and interact to jointly regulate brain injury after SAH. Furthermore, we explored the interrelated molecular mechanisms and targets to provide new ideas for drug synthesis. This review provides novel research directions that can aid in improving the prognosis of patients with SAH.

## Author contributions

JC, KX, and YW designed the study. JC and ML drafted the manuscript. All authors discussed the results and revised the manuscript and read and approved the final manuscript.
